# The prescriptions from Shenghui soup enhanced neurite growth and GAP-43 expression level in PC12 cells

**DOI:** 10.1186/s12906-016-1339-y

**Published:** 2016-09-20

**Authors:** Qi Zhang, Zi-Jian Zhang, Xing-Hua Wang, Jie Ma, Yue-Han Song, Mi Liang, Sen-Xiang Lin, Jie Zhao, Ao-Zhe Zhang, Feng Li, Qian Hua

**Affiliations:** 1 School of Basic Medical Science, Beijing University of Chinese Medicine, No.11 3rd North Ring Road, Chaoyang District, Beijing 100029 People’s Republic of China; 2 Beijing Institute of Traditional Chinese Medicine, Beijing University of Chinese Medicine, No.11 3rd North Ring Road, Chaoyang District, Beijing 100029 People’s Republic of China

**Keywords:** Shenghui soup, Neurite, PC12 cells, GAP-43

## Abstract

**Background:**

Shenghui soup is a traditional Chinese herbal medicine used in clinic for the treatment of forgetfulness. In order to understanding the prescription principle, the effects of “tonifying *qi* and strengthening spleen” group (TQSS) including *Poria cocos* (Schw.) Wolf. and *Panax ginseng* C.A.Mey and “eliminating phlegm and strengthening intelligence” group (EPSI) composed of *Polygala tenuifolia* Willd., *Acorus calamus* L. and *Sinapis alba* L from the herb complex on neurite growth in PC12 cells, two disassembled prescriptions derived from Shenghui soup and their molecular mechanisms were investigated.

**Methods:**

Firstly, CCK-8 kit was used to detect the impact of the two prescriptions on PC12 cell viability; and Flow cytometry was performed to measure the cell apoptosis when PC12 cells were treated with these drugs. Secondly, the effect of the two prescriptions on the differentiation of PC12 cells was observed. Finally, the mRNA and protein expression levels of GAP-43 were analyzed by RT-PCR and western blot, respectively.

**Results:**

“Tonifying *qi* and strengthening spleen” prescription decreased cell viability in a dose-dependent manner, but had no significant effect on cell apoptosis. Meanwhile, it could improve neurite growth and elevate the mRNA and protein expression level of GAP-43. “Eliminating phlegm and strengthening intelligence” prescription also exerted the similar effects on cell viability and apoptosis. Furthermore, it could also enhance cell neurite growth, with a higher expression level of GAP-43 mRNA and protein.

**Conclusion:**

“Tonifying qi and strengthening spleen” and “eliminating phlegm and strengthening intelligence” prescriptions from Shenghui soup have a positive effect on neurite growth. Their effects are related to the up-regulating expression of GAP-43.

**Electronic supplementary material:**

The online version of this article (doi:10.1186/s12906-016-1339-y) contains supplementary material, which is available to authorized users.

## Background

The ability of learning and memory is impaired in neurodegenerative disorders including Alzheimer’s disease [1], Parkinson’s disease [2], Huntington’s disease [3], etc. In addition, many other factors also cause the decline of learning and memory such as stress [4], aging [5], brain injury [6] and sleep deprivation [7]. The population with memory disorder is increasing under these conditions. It poses a huge burden on family and society. Although the mechanism of learning and memory has been reported much at present, effective drugs for improving the memory and cognition are very few.

Synaptic plasticity is the neurochemical foundation of learning and memory. It refers to the activity-dependent modification of the strength or efficacy of synaptic transmission [8]. Plastic changes occur in both structure and function. Accumulating evidence suggests that impairments in synaptic mechanisms contribute to several prominent neuropsychiatric disorders including dementia [9] and extrapyramidal disorders [10]. The structural plasticity of synapse is dependent on various internal and external factors that regulate cytoskeleton components in axis cylinder, growth cone, dendrite and dendritic spines, and then exert the changes in structure [11, 12].

Growth Associated Protein 43 (GAP-43) is a presynaptic protein that is located on the inner surface of axon terminal plasma membrane and participates in signal transduction processes in nerve terminals [13]. The researchers have found high mRNA and protein expression levels of GAP-43 in the developing nervous system or during the regeneration process of damage neurons [14]. Meanwhile, studies have shown that GAP-43 plays a pivotal role in promoting axonal growth and exerting new linkage of axons, and has a close relationship with the development of nervous system, synapse formation as well as nerve regeneration. Therefore, GAP-43 is considered as intrinsic presynaptic determinant for neurite outgrowth and synaptic plasticity [15].

Shenghui soup is a traditional Chinese herbal prescription, which has favorable therapeutic efficacy on treating senile forgetfulness and enhancing memory in clinic [16–18]. It contains nine herbs including *Rehmannia glutinosa* (Gaertn.) DC., *Polygala tenuifolia* Willd., *Cornus officinalis* Siebold & Zucc., *Platycladus orientalis* (L.) Franco., *Acorus calamus* L., *Ziziphus jujuba* Mill., *Poria cocos* (Schw.) Wolf., *Sinapis alba* L. and *Panax ginseng* C.A.Mey. Zhou and colleagues found that Shenghui soup could improve the learning and memory of scopolamine-treated mice [19]. In addition, the ability of learning and memory of fatigue rat was also enhanced by the combination of Shenghui soup and Sini powder [20]. According to the medicinal property theory of traditional Chinese medicine, the nine herbs in the recipe are classified into four categories: “Enriching *yin* and nourishing kidney” group (*Rehmannia glutinosa* (Gaertn.) DC. and *Cornus officinalis* Siebold & Zucc.), “nourishing heart and tranquilizing mind” group (*Ziziphus jujuba* Mill. and *Platycladus orientalis* (L.) Franco.), “tonifying *qi* and strengthening spleen” group (*Poria cocos* (Schw.) Wolf. and *Panax ginseng* C.A.Mey, TQSS), as well as “eliminating phlegm and strengthening intelligence” group (*Polygala tenuifolia* Willd., *Acorus calamus* L. and *Sinapis alba* L., EPSI). In our previous studies, it was found that the tonifying *qi* and strengthening spleen group (*Poria cocos* (Schw.) Wolf. and *Panax ginseng* C.A.Mey) and the eliminating phlegm and strengthening intelligence group (*Polygala tenuifolia* Willd., *Acorus calamus* L. and *Sinapis alba* L.) derived from Shenghui soup have effects on the proliferation of SH-SY5Y cells [21].

PC12 cells, a cell line derived from a pheochromocytoma of rat adrenal medulla, represent a model for neuronal differentiation [22]. Nerve growth factor could induce differentiation of PC12 cells into cholinergic neurons-like cells [23]. Its characteristics include neurite growth and the formation of synapse. Therefore, PC12 cells can be used as a model of synaptic plasticity. In this work, the effects of “tonifying *qi* and strengthening spleen” and “eliminating phlegm and strengthening intelligence” prescriptions on PC12 cells and the possible molecular mechanisms were investigated. Firstly, the cell viability and apoptosis percentage of PC12 cells treated with the test drugs were measured and then the influences of TQSS and EPSI on the PC12 differentiation were observed. At last, the mRNA and protein expression levels of GAP-43 were evaluated with RT-PCR and western blot, respectively.

## Methods

### Reagents

The TQSS (*Poria cocos* (Schw.) Wolf. and *Panax ginseng* C.A.Mey) and EPSI (*Polygala tenuifolia* Willd., *Acorus calamus* L. and *Sinapis alba* L.) prescriptions derived from Shenghui soup were dry powders obtained from Prof. Feng Li Nerve Immunology Laboratory of Beijing University of Chinese Medicine and they were formulated into a concentrated solution with the final concentration of 100 mg/mL (represented by the amounts of crude drug per milliliter sterilized 0.01 M PBS buffer) stored at 4 °C. Membrane filters with pore-size ratings of 0.22 μm were used for sterilization before the experiment.

Dulbecco’s modified Eagle’s medium (DMEM), Fetal Bovine Serum (FBS), Penicillin-Streptomycin, 0.25 % Trypsin-EDTA and Horse Serum (HS) were all purchased from Gibco (Carlsbad, CA, USA). The Cell Counting Kit (CCK-8) was purchased from Dojindo (Kumamoto, Japan). The cell adhesion agent Poly-L-lysine was purchased from Applygen (Beijing, China). Annexin V-FITC cell apoptosis analysis kit was purchased from Beyotime (Jiangsu, China). The EasyScript First-Strand cDNA Synthesis SuperMix kit and 2 × EasyTaq PCR SuperMix kit were purchased from TransGen (Beijing, China). Mouse Anti-GAP-43 monoclonal antibody was purchased from Abcam (Cambridge, UK). Mouse anti-β-actin monoclonal and HRP-conjugated Goat anti-mouse IgG antibodies were purchased from CWBIO (Beijing, China). The polyvinylidene difluoride (PVDF) membrane was purchased from Millipore (Billerica, MA, USA) and the enhanced chemiluminescence (ECL) substrate was purchased from Pierce (Boston, MA, USA).

### The fingerprint of TQSS and EPSI

The TQSS or EPSI extract was were powdered to a homogeneous size by a mill and sieved through a No. 40 mesh sieve. An amount of 1.0 g was extracted with 25 mL of methanol/water (70:30, v/v) in an ultrasonic bath (Eima Ultrasonics Corp., Germany) for 30 min at room temperature. The methanol solution was filtered through a 0.22 μm membrane before injection to the HPLC-MS system for analysis.

The chromatographic separation was performed with a Kromasil 100 C18 column (4.6 × 250 mm i.d., 5 μm) using a Dionex Ultimate 3000 HPLC system (Thermo Fisher Scientific, CA, USA). Acetonitrile and 0.1 % formic acid aqueous solution were used as mobile phase. The flow rate was 1.0 mL/min at 30 °C applied with a linear gradient as follows: 0 min, 5 % B; 2 min, 5 % B; 42 min, 40 % B; 50 min, 55 % B; 55 min, 85 % B; 60 min, 5 % B; 65 min, 5 % B. The column was equilibrated for 5 min before the next injection start. EPSI extract were detected by HPLC with diode array detector and he wavelength was set at 326 nm. It was found the Sinapine thiocyanate (1) at 326 nm in the chromatogram of EPSI extract.

**Fig. 1:**
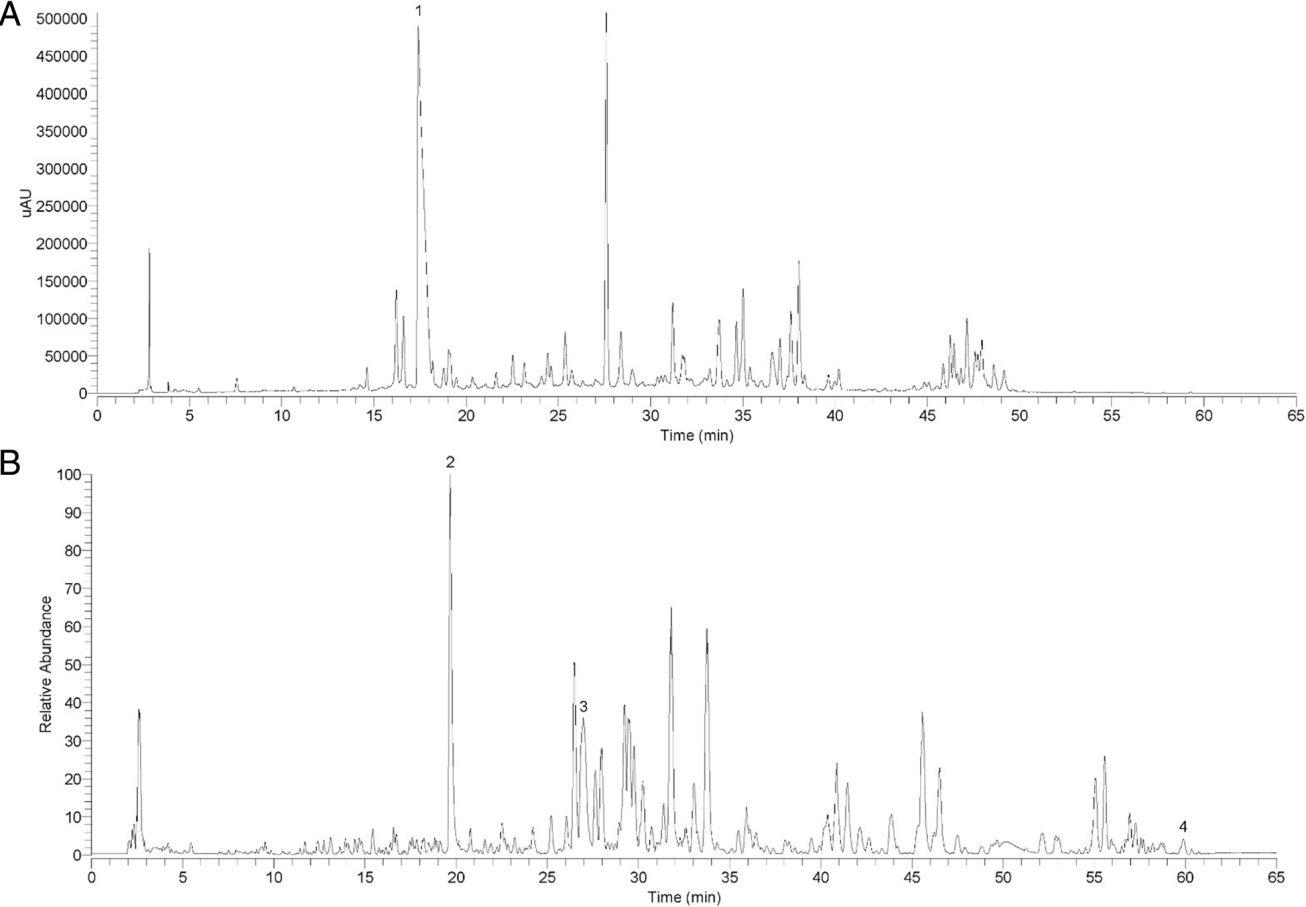
The fingerprint of EPSI and TQSS. **a** EPSI was analyzed by HPLC with diode array detector. 1 represents sinapine thiocyanate. **b** TQSS fingerprint was obtained by high resolution accurate mass spectrometry. 2, 3 and 4 represent ginsenoside Rg1, ginsenoside Rb1 and pachymic acid, respectively

Due to the TQSS extract had a weak UV absorption, we performed the MS to detect the active ingredient. High resolution accurate mass data for TQSS extract analysis was acquired in positive ion modes using a LTQ-Orbitrap (Thermo Fisher Scientific, Germany) via ESI interface. MS parameters were set as follows: spray voltage of 3 kV in positive mode and 2.8 kV in negative mode, heat temperature of 350 °C, sheath gas flow rate of 40 arbitrary units, auxiliary gas of 10 arbitrary units, and capillary temperature of 300 °C. The full scan data were recorded at mass resolving power of 30,000 (FWHM, calculated for m/z 200). As shown in Fig. 1b, the base peaks with Ginsenoside Rg1 (2), Ginsenoside Rb1 (3) and Pachymic acid (4) are labelled in the chromatogram of the TQSS extract.

### Cell lines

PC12 cells were purchased from Cell Resource Center of Peking Union Medical College (which is the headquarter of National Infrastructure of Cell Line Resource, NSTI).

### Cell culture and treatment

PC12 cells were cultured in DMEM supplemented with 5 % heat-inactivated FBS, 10 % heat-inactivated HS, 100 units/mL penicillin, and 100 μg/mL streptomycin sulfate. They were maintained at 37 °C under 95 % air and 5 % CO_2_. Medium renewal was carried out 2–3 times/week. To observe the impact of drugs on neurite growth, PC12 cells were plated onto Poly-L-Lysine-coated 24-well tissue culture plates and the medium was replaced with DMEM containing 1 % heat-inactivated HS and 0.5 % heat-inactivated FBS.

### Cell viability assay

The cell viability was detected with the Cell Counting Kit-8 according to the manufacturer’s instruction. PC12 cells were seeded in flat-bottomed 96-well plates at a density of 5 × 10^4^ cells per well in 100 μL DMEM with 1 % HS and 0.5 % FBS. After 24 h of incubation, the candidate drugs resolved in the medium were added. The final concentrations of TQSS groups were 10, 20, 50, 100, 200, 500 and 2000 mg/L and EPSI groups were 5, 10, 20, 50, 100, 200 and 500 mg/L. Then, the cells were cultured for another 48 h. There were six parallel wells for each concentration. At indicated time point, 10 μL of the CCK-8 solution were added into each well. After 2 h of incubation, the absorbance at 450 nm was measured using a microplate reader (Tecan, Safire, Switzerland). All experiments were performed in triplicate on three separate occasions.

### Apoptosis detection by flow cytometry

Four groups were set in this assay including normal, control, TQSS and EPSI, respectively. The normal group was cultured in DMEM with 5 % heat-inactivated fetal bovine serum (FBS) and 10 % heat-inactivated horse serum (HS). The control group was cultured in DMEM with 0.5 % heat-inactivated FBS and 1 % heat-inactivated HS. The TQSS and EPSI groups were 1000 mg/L and 500 mg/L crude drug in low-serum DMEM, respectively. Firstly, PC12 cells were seeded in 6-well plates at the density of 2 × 10^6^ cells per well. After overnight, the cells were handled as described above. After 48 h treatment, PC12 cells were detached with 0.25 % trypsin-EDTA digestion, collected by centrifugation at 1000 rpm for 5 min and washed with cold PBS. The cells of each group were stained according to the manufacture’s instruction. After that, flow cytometry was performed to determine the apoptosis percentage of PC12 cells on a Beckman Cell Lab Quata SC flow cytometry. Both early apoptotic (Annexin V-positive, PI-negative) and late (Annexin V-positive and PI-positive) apoptotic cells were included in cell death determinations.

### Morphometric analysis of neurite growth

PC12 cells were resuspended in DMEM with 0.5 % heat-inactivated FBS and 1 % heat-inactivated and seeded at the density of 10,000 cells per well in 24-well plates pre-coated with cell adhesion agent Poly-L-lysine. After 24 h culture, the candidate drugs were supplemented into medium. The final concentrations of TQSS group were 250, 500 and 1000 mg/L, and the EPSI group were 100, 200 and 500 mg/L. Three parallel samples were designed in each concentration. For additional 48 h treatment, the neurites were observed with the microscope.

The length of neurite analysis was conducted as follows: Firstly, two random fields were examined in each well after 48 h of treatment by using a digital camera (Canon, Krefeld, Germany) linked to an inverted microscope with phase contrast illumination (Olympus, Hamburg, Germany) at 200× magnifications. Secondly, the number and length of the neurites in the taken pictures were measured using Image J software. The length of the longest neurite of differentiated cells was calculated as the ratio of measurable neurite length and the number where at least 100 neurites were counted in each group.

### The mRNA expression of GAP-43 by RT-PCR

PC12 cells were resuspended in a low-serum DMEM and seeded at the density of 1 × 10^6^ cells per well in 6-well plates coated with cell adhesion agent Poly-L-lysine. The drugs (TQSS and EPSI) were supplemented to the medium. The final concentrations of TQSS were 250, 500 and 1000 mg/L and EPSI were 100, 200 and 500 mg/L. After 48 h incubation, the samples were collected. Total RNA of each sample was extracted using Trizol reagent, and then 0.8 % agarose gel electrophoresis was used to assess the RNA quality. The concentration of total RNA was measured with Thermo NanoDrop 2000 and the purity was estimated with the ratio of OD_260_ and OD_280_.

The reverse transcription reaction was conducted according to manufacturer’s protocol. The equal total RNA of each sample was loaded to prepare the first strand cDNA. Then, the cDNA product was subsequently employed as the PCR template to amplify the target gene GAP-43 and 18S ribosomal RNA as an internal standard. The primers used in the PCR amplification were synthesized by Sangon Biotech (Shanghai, China) and listed in Table 1. After PCR amplification, the products were analyzed with 2 % agarose gel electrophoresis. The optical density of GAP43 and 18S rRNA of each sample in four groups were quantified with Quantity One software (Bio-Rad, USA) and the ratio of the two genes were calculated. The percentages of drug groups and control group were plotted where the control group was normalized to 100 %.

**Table 1:** RT-PCR primers

Gene	Primer	Sequence (5′ to 3′)	Length (bp)
GAP-43	Forward	CCGAGGCTGACCAAGAACA	122
	Reverse	TGAGCAGGACAGGAGAGGAA	
18S rRNA	Forward	ACACGGACAGGATTGACAGA	238
	Reverse	GGACATCTAAGGGCATCACAG	

### The protein expression of GAP-43 by western blot

PC12 cells were resuspended in a low-serum DMEM and seeded at the density of 4 × 10^6^ cells per well in 60 mm petri dish coated with cell adhesion agent Poly-L-lysine. The candidate prescriptions were added as described in 2.5. After a 48 h treatment, RIPA lysis buffer containing protease inhibitor was applied to extract total proteins for each sample, and protein concentration was determined using BCA Protein Assay Kit (Pierce, Thermo Scientific). The GAP-43 protein level was detected by western blot with β-actin as a loading control. Protein sample of 30 μg was loaded on 12 % SDS-PAGE gels with a constant electrophoresis at 100 V for 2.5 h. After that, the protein in the gel was transferred to PVDF membranes with a constant electrophoresis at 300 mA for 60 min and blocked with 5 % non-fat dry milk for 1.5 h. Rat anti-GAP-43 monoclonal antibody (1:2000) was incubated overnight at 4 °C. After triplicate washed with PBST (0.1 % Tween 20 solved in the PBS), the membranes were incubated with goat anti-rat IgG-HRP (1:4000) at 37 °C for 1.5 h. Finally, the membranes were developed with enhanced chemiluminescence (ECL) substrate and exposed to X-ray film. The bands were analyzed with Quantity one software (Bio-Rad, USA).

### Statistical analysis

All experiments were performed at least three times. Data are presented as mean ± standard deviation (SD). Statistical analyses for testing of significance were analyzed using one-way analysis of variance (ANOVA) and post hoc Bonferroni tests were done where appropriate. A value of *p* < 0.05 was considered to be statistically significant, and the analysis was carried out using SPSS Version 21.0 software (IBM SPSS Inc., Chicago, Illinois, USA).

## Results

### The TQSS and EPSI treatment inhibited the viability of PC12

**Fig. 2:**
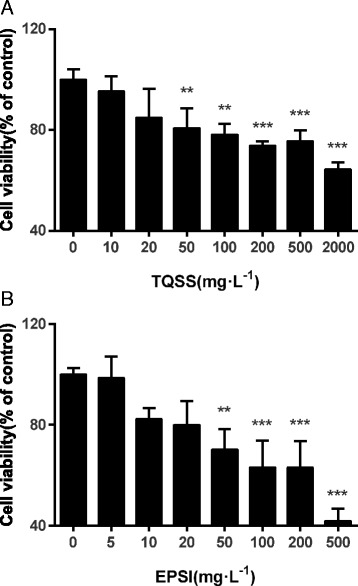
The TQSS and EPSI drugs inhibited the viability of PC12 cells. **a** The cell viability of PC12 cells incubated for 48 h with TQSS derived from Shenghui soup at different concentrations (10, 20, 50, 100, 200, 500 and 2000 mg/L). **b** PC12 cells were treated with different concentrations (5, 10, 20, 50, 100, 200 and 500 mg/L) of EPSI derived from Shenghui soup for 48 h. Data are expressed as mean ± SD, *n* = 3, *p < 0.05, **p < 0.01, ***p < 0.001 versus control without drugs

In order to investigate the effect of the separated prescriptions (TQSS group and EPSI group) from Shenghui soup on PC12 viability, different concentrations of the two prescriptions were supplemented to the low serum media. After 48 h of incubation, it was found that there was a dose-dependent decreasing of cell viability under both TQSS and EPSI exposure. Low levels of TQSS and EPSI treatments (<50 mg/L) had no significant reduction on the viability. While the concentrations of TQSS drug were equal or greater than 50 mg/L, the cell viability was significantly inhibited (50 mg/L, *p* = 0.034; 100 mg/L, *p* = 0.031; 200 mg/L, *p* = 0.019; 500 mg/L, *p* = 0.016; 2000 mg/L, *p* = 0.011) (Fig. 2a). Similarly, PC12 cells viabilities showed significant declines after treated with 50 mg/L, 100 mg/L, 200 mg/L and 500 mg/L EPSI drug for 48 h (Fig. 2b).

To verify the result, we measured the cell viability with MTT assay. PC12 cells were growth in another density with 7000/well. The same concentrations of TQSS and EPSI were used to treat PC12 cells. The similar results were obtained (Additional file 1: Figure S1).

### High doses of TQSS and EPSI recipes have no significant effect on the apoptosis of PC12 cells

**Fig. 3:**
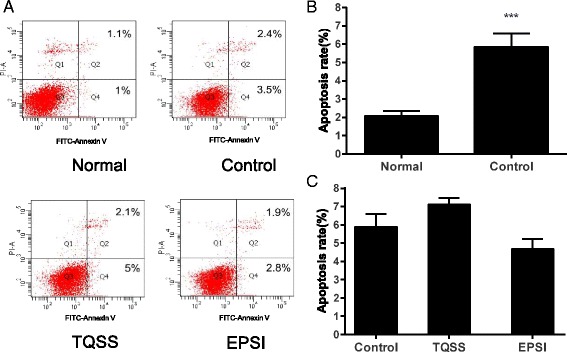
The apoptosis of PC12 cells was not affected by the TQSS and EPSI formula. **a** The representative apoptotic pictures of PC12 cells detected by flow cytometry. The normal group and control group represent the cells cultured with 15 % serum DMEM and 1.5 % serum DMEM, respectively. The TQSS and EPSI groups stand for the apoptosis rates of PC12 cells treated with 1000 mg/L TQSS and 500 mg/L EPSI in 1.5 % serum DMEM, respectively. **b** The statistical analysis for control group and normal group. **c** The statistical analysis of the TQSS and EPSI treated group and the control group. Data are expressed as mean ± SD, *n* = 3, ***p < 0.001 versus normal

Subsequently, the apoptosis of PC12 cells treated with the two separated prescriptions from Shenghui soup was determined. Considering that low concentration of serum could induce differentiation of PC12 cells, the normal group was also assessed, in which the cells were cultured in DMEM containing 10 % HS and 5 % FBS. As shown in Fig. 3a and b, the average of apoptotic percentages (Q2 + Q4) in the control group was significantly higher than that in the normal group (The normal group was 2.1 % ± 0.21, and the control group was 5.9 % ± 0.59, *p* = 0.033). In addition, when treated with 1000 mg/L TQSS drug, the apoptotic percentage of PC12 cells was 7.1 % ± 0.28. And after incubation with 500 mg/L EPSI, the average apoptosis rate was 4.7 % ± 0.44. No significant differences were detected between the two drug groups and the control group (TQSS vs control, *p* = 0.466; EPSI vs control, *p* = 0.608). It was suggested that the TQSS and EPSI did not promote the apoptosis of PC12 cultured in the medium with low serum. To conform this result, The TUNEL assay, a more sensitive method to detect early apoptosis cells, was carried with the same experiment pattern. It was shown that the apoptosis rates of TQSS and EPSI groups have no obvious differences compared with control group (Additional file 1: Figure S2).

### High concentration of TQSS and EPSI recipes enhance the neurite growth of PC12 cells

**Fig. 4:**
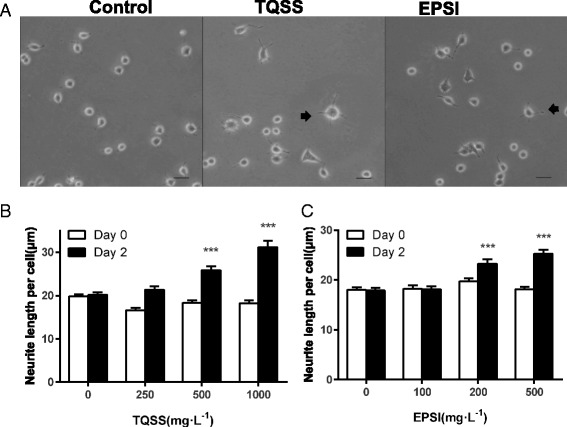
The TQSS and EPSI prescriptions enhanced the neurite growth in PC12 cells. **a** The morphology of PC12 cells treated with 1000 mg/L TQSS and 500 mg/L EPSI. The PC12 cells were incubated with the candidate drugs for 48 h and then they were observed and photographed with the phase contrast microscope (scale bar = 50 μm). **b** The neurite length average of PC12 cells treated with 1000 mg/L TQSS from Shenghui soup. **c** The statistical analysis of neurite length of PC12 cells treated with 500 mg/L EPSI. Data are expressed as mean ± SD, ***p < 0.001 versus zero-day control

The effects of TQSS and EPSI prescriptions on the differentiation of PC12 cells were investigated in this section. Firstly, the cellular morphology of drug-treated groups was observed and photographed. Secondly, the neurite length of PC12 cells were measured and analyzed. It is shown that TQSS prescription (1000 mg/L) and EPSI prescription (500 mg/L) could promote neurite outgrowth compared with control group (Fig. 4a). The cells in the control group were like small dot. After drug stimulation, neurites started to elongate around the cell body and part of them presented irregular triangle shapes. Furthermore, according to the neurite length and cell number, the neurite length per cell for each group was calculated. As shown in Fig. 4b, PC12 cells were treated with TQSS drug at three concentrations (250, 500 and 1000 mg/L). The neurite length per cells before (Day 0) and after (Day 2) were plotted for each concentration. It was found that the average neurite length of PC12 without drug intervention changed little after 48 h of incubation. However, the neurite length per cell significantly increased when PC12 cells were treated with 500 mg/L (*n* > 100, *p* = 0.001) and 1000 mg/L TQSS (*n* > 100, *p* = 0.001). Although there was no significant difference at the concentration of 250 mg/L, the neurite growth was observed to be increased in a dose-dependent manner. Similar result was obtained with EPSI separated prescription from Shenghui soup. The mean of neurite length of PC12 cells increased significantly at 500 mg/L and 1000 mg/L after incubation of two days with drugs (*n* > 100, *p* = 0.008) (Fig. 4c). All these observations indicated that the two separated prescriptions from Shenghui soup, namely TQSS and EPSI, could promote the differentiation of PC12 cells.

### The disassembled formulas from Shenghui soup enhanced mRNA expression of GAP-43 of PC12 cells

**Fig. 5:**
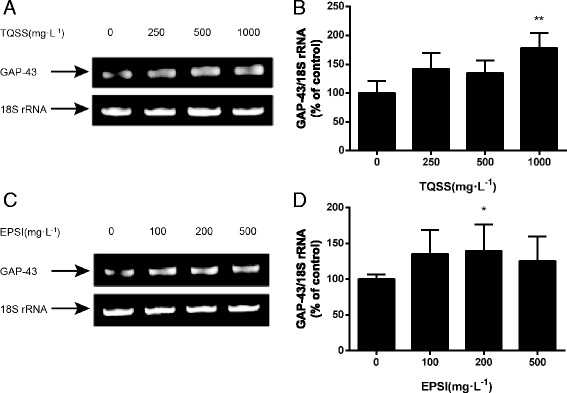
The mRNA level of GAP-43 increased after the TQSS and EPSI treatment. **a** The representative graphic of GAP-43 mRNA expression level analyzed by agarose gel electrophoresis. The lanes were loaded with samples from control group without drugs, 250, 500 and 1000 mg/L TQSS. **b** The GAP-43 mRNA expression for three independent experiments were analyzed and plotted. The gel image (**c**) and column chart (**d**) for EPSI group are shown at the bottom and the EPSI concentrations are 100, 250 and 500 mg/L, respectively. Data are expressed as mean ± SD, *n* = 3, *p < 0.05, **p < 0.01 versus the group without drugs

To further explore the molecular mechanism of enhanced neurite growth induced by TQSS and EPSI prescriptions from Shenghui soup, we sought to measure the mRNA expression level of GAP-43 that is a growth-associated protein located in the cellular membrane by reverse transcription polymerase chain reaction (RT-PCR) and agarose gel electrophoresis. The results showed that the mRNA level of GAP-43 had a rapid increase when treated with different concentrations of TQSS drug (250, 500 and 1000 mg/L) (Fig. 5a). The image analysis showed that the mean optical density of 1000 mg/L treated group was significantly larger than that of the control group without drugs (*p* = 0.027) (Fig. 5b). The same experiment was performed for the EPSI. It was found that the GAP43 expression of PC12 cells was elevated after herbal intervention at 100, 200 and 500 mg/L (Fig. 4c). The statistical data stated that the GAP-43 mRNA expression increased significantly when the EPSI concentration was 200 mg/L. It was revealed that TQSS prescription (1000 mg/L) and EPSI prescription (200 mg/L) from Shenghui soup significantly up-regulated the mRNA expression level of GAP-43.

### The GAP-43 protein expression level increased when treated with TQSS and EPSI

**Fig. 6:**
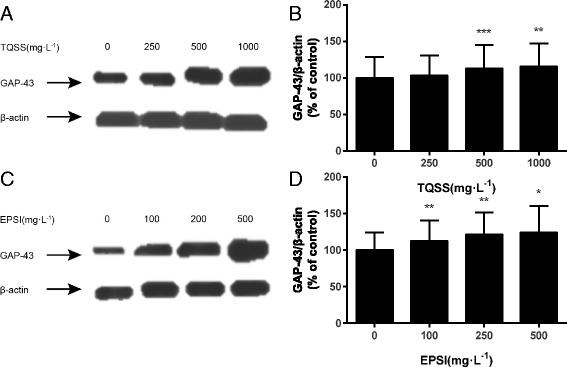
The TQSS and EPSI increased the GAP-43 protein expression of PC12 cells. The representative image of GAP-43 expression of PC12 cells treated with TQSS (**a**) and EPSI (**c**). The TQSS group is loaded with PC12 cells treated with 0, 250, 500 and 1000 mg/L concentration. The concentrations of EPSI group are 0, 100, 200 and 500 mg/L. The statistical results of the two drug groups are on the *right* (**b** and **d**). Data are expressed as mean ± SD, *n* = 3, *P < 0.05, **P < 0.01, ***p <0.001 versus control with no drugs

For further research, the GAP-43 protein level of PC12 cells was also measured by western blot. Like the RT-PCR assay, PC12 cells were treated by TQSS (250, 500 and 100 mg/L) and EPSI (100, 200 and 500 mg/L) at the given concentrations, respectively. The results showed that the GAP-43 expression of PC12 cells treated by TQSS components increased significantly at the concentrations of 500 mg/L (*p* = 0.043) and 1000 mg/L (*p* = 0.036) compared with the control group (Fig. 6a and b). No obvious difference of GAP expression was observed between PC12 cells incubated with 250 mg/L TQSS and the control cells. Moreover, the EPSI group shared the same situation with the TQSS. It enhanced the expression of GAP-43 in a dose-dependent manner with the GAP expressions of PC12 treated with EPSI having significant increase at all the used concentrations (Fig. 5c and d).

## Discussion

In this study, our results showed that the viability of PC12 cells treated with TQSS and EPSI, with two separated prescriptions from Shenghui soup, decreased when cultured in the low serum medium. However, there are no significant differences in the apoptotic rate of TQSS and EPSI groups compared with the low serum group. It was observed that the two formulas derived from Shenghui soup would not induce cell apoptosis and had no toxic effect on PC12 cells. Thus, the inhibition effect on PC12 viability may have other causes. It has been reported that the low serum culture may inhibit the cell proliferation and promote differentiation [24]. Our cell cycle result showed that G1 cell percentage of PC12 has a significant increase when culture in low serum. However, TQSS and EPSI treatment didn’t induce G1 arrest (data not shown).

Therefore, the effects of the neurite formation of PC12 cells were taken into account.

After two days of incubation with TQSS and EPSI, the morphology of PC12 cells cultured in the low serum medium had no large changes. They were still round and few neurites were extended from the cell body. However, the neurites outgrowth could be seen obviously in the herbal medicine groups and some of the cells had presented the neuron-like shape. The differentiation process of PC12 cells in vitro usually needs at least three consecutive days or more [25]. All these findings along with the previous reports demonstrate that the two formulas from Shenghui soup can efficiently promote differentiation and neurite extension of PC12 cells. Moreover, it can be explained that they may lead to the viability reduction through inducing the differentiation of PC12 cells.

Furthermore, RT-PCR and western blot results showed the TQSS and EPSI formula could increase the GAP-43 expression in PC12 cells, and the EPSI group played a better pharmacological effect than TQSS group. It has been found that total ginsenoside, the active component of ginseng in the TQSS group can promote the neurite outgrowth and expression of GAP-43 in PC12 cells [26]. Pi and colleagues [27] reported that zu, the major active ingredient of polygala, has nutrition effect on new rat cortical neurons and promotes neurite outgrowth. Therefore, this may be the reason why TQSS and EPSI prescriptions can promote the neurite outgrowth. However, we cannot determine whether other monomer components may have effects on synaptic plasticity.

The nervous system is dynamic variable network. Brain development and functional establishment are accompanied by morphological and molecular changes in neurons [28]. Neuron specific protein GAP-43 is one of the important constituents involved in basic nervous system process and is highly expressed during early stages of development [29]. GAP-43 is required for axonal growth cone guidance, and in the mature neurons, GAP-43 localized in the presynaptic region is involved in synaptic plasticity. Numerous studies have demonstrated that overexpression of GAP-43 protein in the nervous system of adult transgenic mice is accompanied by increased learning capacity [30, 31]. We do believe that the two disassembled prescriptions from Shenghui soup, TQSS and EPSI, can improve learning and memory by enhancing the synaptic plasticity.

Shenghui soup is a traditional Chinese herbal medicine composed of nine herbal drugs in fixed proportions. It has been used to improve learning and memory in clinic. In order to study its mechanism, it is divided into four prescriptions that include enriching *yin* and nourishing kidney group (*Rehmannia glutinosa* (Gaertn.) DC. and *Cornus officinalis* Siebold & Zucc.), nourishing heart and tranquilizing mind group (*Ziziphus jujuba* Mill. and *Platycladus orientalis* (L.) Franco.), tonifying *qi* and strengthening spleen group (*Poria cocos* (Schw.) Wolf. and *Panax ginseng C.A.Mey*), and eliminating phlegm and strengthening intelligence group (*Polygala tenuifolia* Willd., *Acorus calamus* L. and *Sinapis alba* L.). As the nourishing heart and tranquilizing mind group is difficult to dissolve into water, it has been excluded in some experiments. Our previous studies have shown that the enriching *yin* and nourishing kidney group could significantly decrease neurite outgrowth and inhibit the differentiation of PC12 cells [21]. This suggests that Shenghui Soup’s enhanced learning and memory is a comprehensive outcome of herbs with different medicinal properties in the formula. The enriching *yin* and nourishing kidney prescriptions may reduce the synaptic plasticity, whereas the tonifying *qi* and strengthening spleen formula and eliminating phlegm and strengthening intelligence recipe enhance the synaptic plasticity. Although the enriching *yin* and nourishing kidney prescription inhibits the neuronal differentiation, it has the neurotrophic and neuroprotective effect. In Shenghui soup, three combinations coexist. They may play different roles jointly to participate in the learning and memory improvement.

## Conclusion

In summary, the tonifying *qi* and strengthening spleen formula and eliminating phlegm and strengthening intelligence recipe derived from Shenghui soup can promote the extension of neurite, enhance the differentiation of PC12 cells, and increase the expression of GAP-43. It means that they can improve the synaptic plasticity. Besides, they will not induce the apoptosis of PC12 cells. In brief, the results of the current study not only further strengthen our understanding of the molecular mechanism that how Shenghui soup improves learning and memory, but also have provided the evidences of its safety for clinical application, which leads us to look for more monomer composition derived Shenghui soup for the treatment of neurological diseases.

## Additional files

Additional file 1: Figure S1. The TQSS and EPSI drugs suppressed the viability of PC12 cells. (A) The viability of PC12 cells was measured with MTT after incubated for 48 h with TQSS derived from Shenghui soup at different concentrations (10, 20, 50, 100, 200, 500 and 2000 mg/L). (B) The relative viability of PC12 cells treated with different concentrations (5, 10, 20, 50, 100, 200 and 500 mg/L) of EPSI derived from Shenghui soup for 48 h. Data are expressed as mean ± SD, *n* = 3, ***p* < 0.01, ****p* < 0.001 versus control without drugs. **Figure S2.** Little apoptosis of PC12 cells treated with TQSS and EPSI occurred. (A) The representative TUNEL pictures of PC12 cells detected by flow cytometry. The normal group and control group represent the cells cultured with 15 % serum DMEM and 1.5 % serum DMEM, respectively. The TQSS and EPSI groups stand for the apoptosis rates of PC12 cells treated with 1000 mg/L TQSS and 500 mg/L EPSI in 1.5 % serum DMEM, respectively. (B) The statistical analysis for control group, normal group TQSS and EPSI groups Data are expressed as mean ± SD, *n* = 3, ****p* < 0.001 versus normal. (PDF 152 kb) Additional file 2: Supplementary data. (DOCX 15 kb)

## Abbreviations

ANOVA: Analysis of variance
CCK-8: Cell counting kit
DMEM: Dulbecco’s modified eagle’s medium
ECL: Enhanced chemiluminescence
EPSI: Eliminating phlegm and strengthening intelligence
FBS: Fetal bovine serum
GAP-43: Growth associated protein 43
HS: Horse serum
PAGE: Polyacrylamide gel electrophoresis
PBS: Phosphate-buffered saline
PVDF: Polyvinylidene difluoride
SD: Standard deviation
SDS: Sodium dodecyl sulfate
TCM: Traditional Chinese medicine
TQSS: Tonifying qi and strengthening spleen
