# Supplementary figures and images for: The prescriptions from Shenghui soup enhanced neurite growth and GAP-43 expression level in PC12 cells
Source: BMC Complement Altern Med. 2016 Sep 20;16:369. doi: 10.1186/s12906-016-1339-y (PMC5029060; doi:10.1186/s12906-016-1339-y)

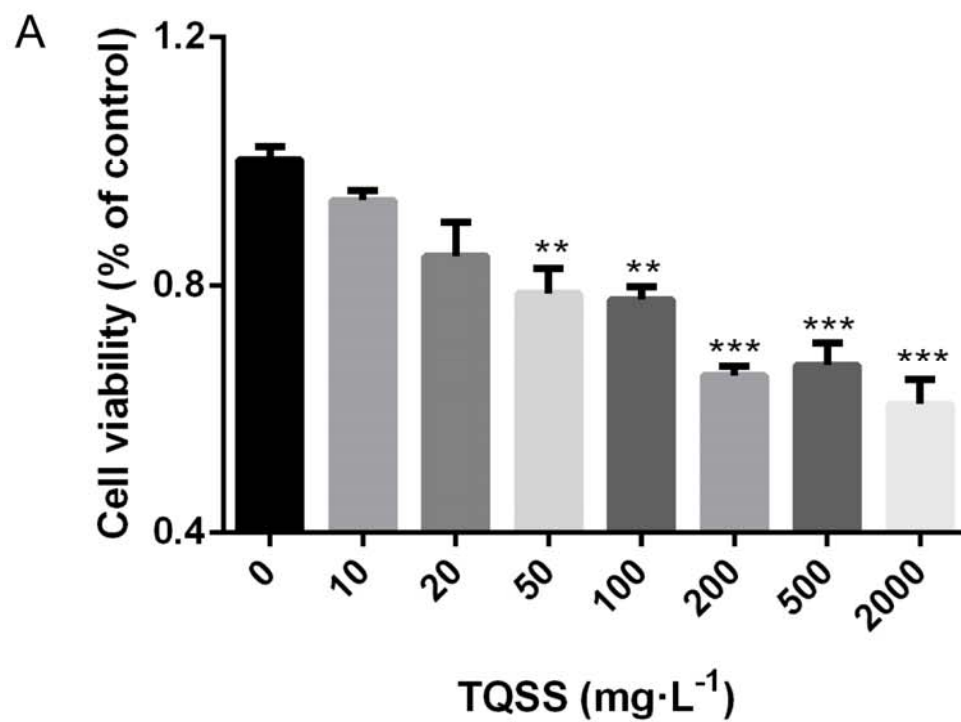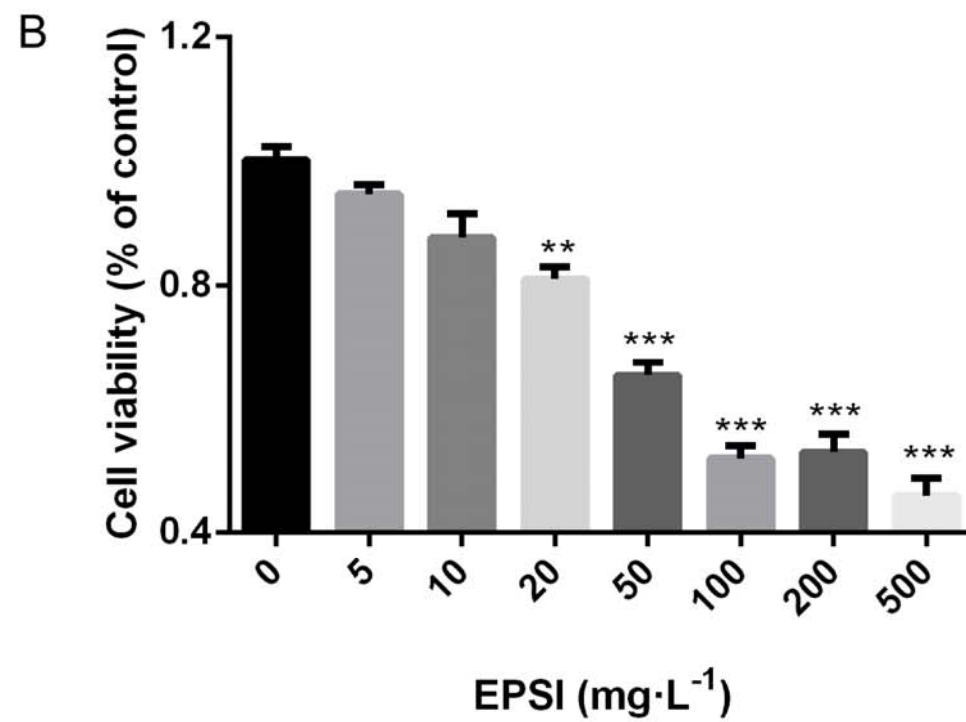

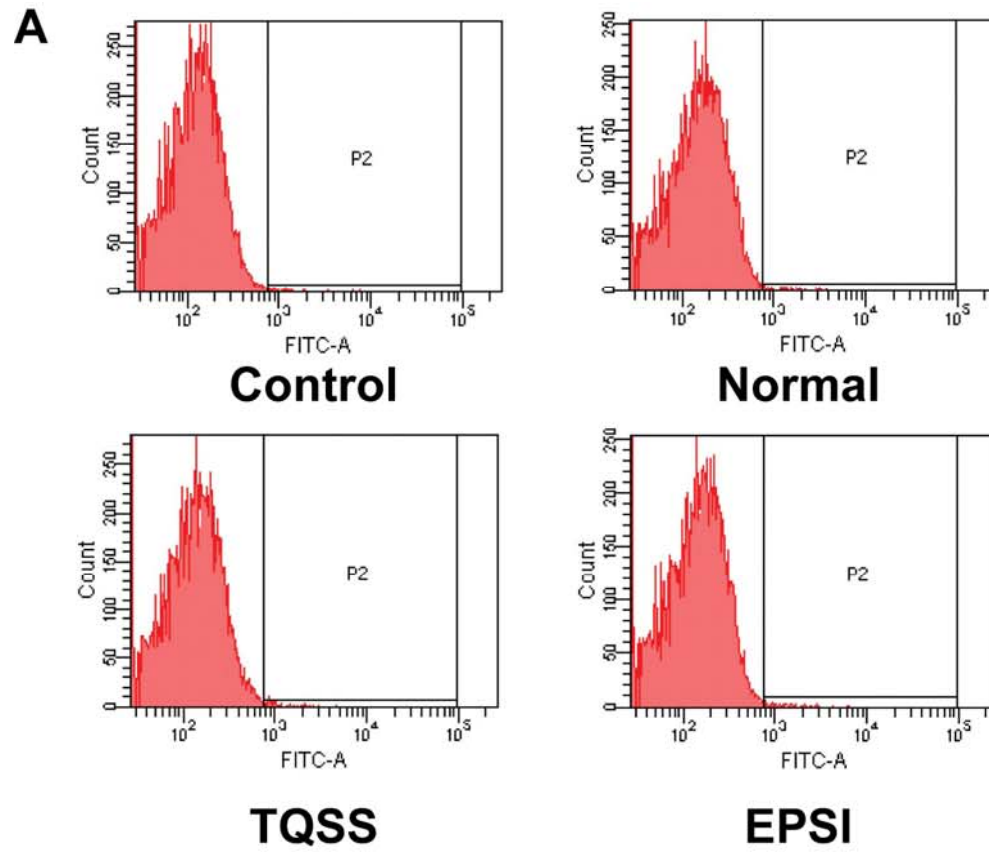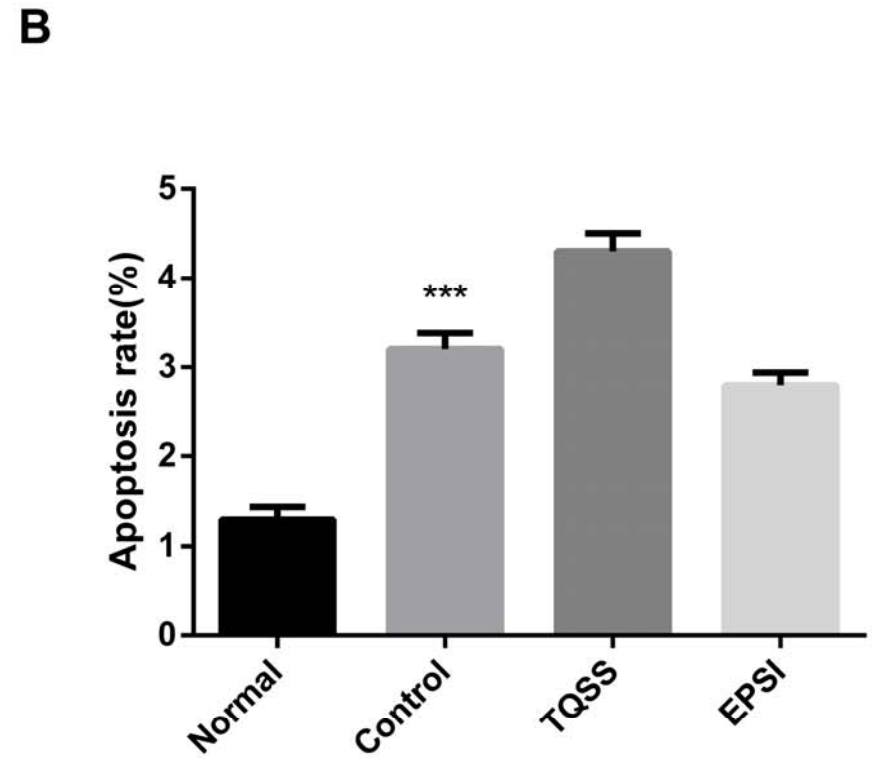

Supplement: Additional file 1: Figure S1. — The TQSS and EPSI drugs suppressed the viability of PC12 cells. (A) The viability of PC12 cells was measured with MTT after incubated for 48 h with TQSS derived from Shenghui soup at different concentrations (10, 20, 50, 100, 200, 500 and 2000 mg/L). (B) The relative viability of PC12 cells treated with different concentrations (5, 10, 20, 50, 100, 200 and 500 mg/L) of EPSI derived from Shenghui soup for 48 h. Data are expressed as mean ± SD, n = 3, **p < 0.01, ***p < 0.001 versus control without drugs. Figure S2. Little apoptosis of PC12 cells treated with TQSS and EPSI occurred. (A) The representative TUNEL pictures of PC12 cells detected by flow cytometry. The normal group and control group represent the cells cultured with 15 % serum DMEM and 1.5 % serum DMEM, respectively. The TQSS and EPSI groups stand for the apoptosis rates of PC12 cells treated with 1000 mg/L TQSS and 500 mg/L EPSI in 1.5 % serum DMEM, respectively. (B) The statistical analysis for control group, normal group TQSS and EPSI groups Data are expressed as mean ± SD, n = 3, ***p < 0.001 versus normal. (PDF 152 kb) [file 12906_2016_1339_MOESM1_ESM.pdf]
