# Supplementary material for: The prescriptions from Shenghui soup enhanced neurite growth and GAP-43 expression level in PC12 cells
Source: BMC Complement Altern Med. 2016 Sep 20;16:369. doi: 10.1186/s12906-016-1339-y (PMC5029060; doi:10.1186/s12906-016-1339-y)
Supplement: Additional file 2: — Supplementary data. (DOCX 15 kb) [file 12906_2016_1339_MOESM2_ESM.docx]

Supplementary data

**Methods**

MTT assay

The cell viability was also determined with the MTT (M-0283, Sigma, USA) PC12 cells were seeded in flat-bottomed 96-well plates at a density of 7×10^3^ cells per well in 200 μL DMEM with 1% HS and 0.5% FBS. After 24 h incubation, the candidate drugs resolved in the medium were added. The final concentrations of TQSS groups were 10, 20, 50, 100, 200, 500 and 2000 mg/L and EPSI groups were 5, 10, 20, 50, 100, 200 and 500 mg/L. Then the cells were cultured for another 48 h. There were three parallel wells for each concentration. At indicated time point, 20 μL of the MTT solution were added into each well. After 4 h incubation, remove cultures from incubator and dissolve the resulting MTT formazan crystals by 200 μL DMSO, the absorbance at 570 nm was measured using a microplate reader. All experiments were performed in triplicate on three separate occasions.

TUNEL assay

Similar to the apoptosis assay, there are four groups in this assay including normal, control, TQSS and EPSI, respectively. The normal group cells were cultured in DMEM with 5% heat-inactivated fetal bovine serum (FBS) and 10% heat-inactivated horse serum (HS). The control group was cultured in DMEM with 0.5% heat-inactivated FBS and 1% heat-inactivated HS. In the TQSS and EPSI groups, 1000 mg/L and 500 mg/L crude drug in low-serum DMEM were used to treat PC12 cells respectively. First, PC12 cells were seeded in 6-well plates at the density of 2 ×10^6^ cells per well. After overnight, the cells were handled as described above. After 48 h treatment, PC12 cells were collected by centrifugation at 1,000 rpm for 5 min and washed with cold PBS. The cells of each group were fixed by 4% paraformaldehyde for 60 min. Cells were washed twice with PBS and suspended with 0.1% Triton X-100 in 0.1% sodium citrate for 2 min. Cells were washed twice with PBS and resuspended in 50 μL/well TUNEL reaction mixture (C1008, Beyotime Biotechnology, China). Then they were incubated for 60 min at 37°C in a humidified atmosphere in the dark. Wash samples twice in PBS and transfer cells into a tube with final volume of 250 μL in PBS. Flow cytometry was performed to determine the apoptosis percentage of PC12 cells on a Beckman Cell Lab Quata SC flow cytometry.

**Figure legends**

**Fig. S1**. The TQSS and EPSI drugs suppressed the viability of PC12 cells. (A) The viability of PC12 cells was measured with MTT after incubated for 48 h with TQSS derived from Shenghui soup at different concentrations (10, 20, 50, 100, 200, 500 and 2000 mg/L). (B) The relative viability of PC12 cells treated with different concentrations (5, 10, 20, 50, 100, 200 and 500 mg/L) of EPSI derived from Shenghui soup for 48 h. Data are expressed as mean ± SD, n=3, **p<0.01, ***p<0.001 versus control without drugs.

**Fig. S2.** Little apoptosis of PC12 cells treated with TQSS and EPSI occurred

(A) The representative TUNEL pictures of PC12 cells detected by flow cytometry. The normal group and control group represent the cells cultured with 15% serum DMEM and 1.5% serum DMEM, respectively. The TQSS and EPSI groups stand for the apoptosis rates of PC12 cells treated with 1000 mg/L TQSS and 500 mg/L EPSI in 1.5% serum DMEM, respectively. (B) The statistical analysis for control group, normal group TQSS and EPSI groups Data are expressed as mean ± SD, n=3, ****p*<0.001 versus normal
